# Euthanasia for people with psychiatric disorders or dementia in Belgium: analysis of officially reported cases

**DOI:** 10.1186/s12888-017-1369-0

**Published:** 2017-06-23

**Authors:** Sigrid Dierickx, Luc Deliens, Joachim Cohen, Kenneth Chambaere

**Affiliations:** 1 End-of-Life Care Research Group, Vrije Universiteit Brussel (VUB) & Ghent University, Laarbeeklaan 103, 1090 Brussels, Belgium; 2 Department of Medical Oncology, Ghent University Hospital, Ghent, Belgium

**Keywords:** End-of-life care, Euthanasia, Health policy, Medical decision making, Psychiatric disorders, Dementia

## Abstract

**Background:**

Euthanasia for people who are not terminally ill, such as those suffering from psychiatric disorders or dementia, is legal in Belgium under strict conditions but remains a controversial practice. As yet, the prevalence of euthanasia for people with psychiatric disorders or dementia has not been studied and little is known about the characteristics of the practice. This study aims to report on the trends in prevalence and number of euthanasia cases with a psychiatric disorder or dementia diagnosis in Belgium and demographic, clinical and decision-making characteristics of these cases.

**Methods:**

We analysed the anonymous databases of euthanasia cases reported to the Federal Control and Evaluation Committee Euthanasia from the implementation of the euthanasia law in Belgium in 2002 until the end of 2013. The databases we received provided the information on all euthanasia cases as registered by the Committee from the official registration forms. Only those with one or more psychiatric disorders or dementia and no physical disease were included in the analysis.

**Results:**

We identified 179 reported euthanasia cases with a psychiatric disorder or dementia as the sole diagnosis. These consisted of mood disorders (*N* = 83), dementia (*N* = 62), other psychiatric disorders (*N* = 22) and mood disorders accompanied by another psychiatric disorder (*N* = 12). The proportion of euthanasia cases with a psychiatric disorder or dementia diagnosis was 0.5% of all cases reported in the period 2002–2007, increasing from 2008 onwards to 3.0% of all cases reported in 2013. The increase in the absolute number of cases is particularly evident in cases with a mood disorder diagnosis. The majority of cases concerned women (58.1% in dementia to 77.1% in mood disorders). All cases were judged to have met the legal requirements by the Committee.

**Conclusions:**

While euthanasia on the grounds of unbearable suffering caused by a psychiatric disorder or dementia remains a comparatively limited practice in Belgium, its prevalence has risen since 2008. If, as this study suggests, people with psychiatric conditions or dementia are increasingly seeking access to euthanasia, the development of practice guidelines is all the more desirable if physicians are to respond adequately to these highly delicate requests.

**Electronic supplementary material:**

The online version of this article (doi:10.1186/s12888-017-1369-0) contains supplementary material, which is available to authorized users.

## Background

The practice of assisted dying is increasingly being discussed in a growing number of countries and is regarded more and more as an acceptable last-resort option for those suffering from severe and irreversible diseases [1]. While assisted dying legislation is restricted to those with terminal illness and a limited life expectancy due to somatic disorder in some US states and Canada, assisted dying for people who are not terminally ill, such as those suffering from psychiatric illness or early stage dementia, is legal in the Netherlands, Belgium and Luxembourg [2].

The Belgian Act on Euthanasia stipulates substantive and procedural requirements that must be met for euthanasia to be legally performed [3]. As for the substantive criteria, the request for euthanasia must be voluntary, well considered, repeated and not the result of any external pressure. Moreover, the person should be legally competent at the moment of expressing the request. Furthermore, the person must be in a medically futile condition of constant and unbearable physical or psychological suffering resulting from a serious disorder with no reasonable treatment alternatives or therapeutic perspective.

Some specific medical and ethical issues arise regarding these substantive requirements when evaluating the euthanasia request of a person suffering from a psychiatric disorder or dementia. To be able to express a voluntary and well-considered euthanasia request, the person must have sufficient insight into the illness and prognosis and have the capacity to make treatment decisions. In people with a psychiatric disorder or dementia diagnosis, this capacity may be impaired; the desire to die can also be a symptom of the disease [4–10]. Furthermore, the irreversibility of psychiatric disorders is often questioned since the course of these disorders may fluctuate and can be hard to predict, and prognosis is often uncertain [9, 11, 12].

Procedural requirements include the consultation of a second independent physician and of a third physician if the patient is not expected to die in the foreseeable future. Since those who request euthanasia because of unbearable suffering caused by a psychiatric condition or dementia generally have a longer life expectancy, consultation of a third physician - who should be an expert in the disease according to the law, i.e. a psychiatrist - is required. Moreover, a one-month waiting period is required in these cases between the written request and the performance of euthanasia. Afterwards, physicians must report all cases to the Federal Control and Evaluation Committee on Euthanasia for review.

Although several studies have examined Belgian euthanasia practice both before and after legalization in 2002 [13–17], little is known about the prevalence and characteristics of euthanasia for psychiatric disorders and dementia. In the Committee’s biennial summary reports on all reported euthanasia cases, one group is identified as ‘neuropsychiatric disorders’ [18], but the reports do not mention the precise diagnosis and characteristics of these cases. Recently the popular media have been reporting on high-profile cases involving people with psychiatric disorders. Since people with psychiatric illnesses or dementia are often considered to be an extremely vulnerable patient group, evaluation and monitoring of the euthanasia practice for these persons is vital.

This study aims to describe the trends in reported euthanasia cases with a psychiatric disorder or dementia diagnosis and their characteristics. Only those with one or more psychiatric disorders or dementia and no physical disease are included in the analysis. We will address the following research questions: how has the number of reported euthanasia cases of people with psychiatric disorders or dementia changed between 2002 and 2013, what are the demographic and clinical characteristics of people with psychiatric disorders or dementia who have received euthanasia and what are the characteristics of the decision-making process in reported euthanasia cases of people with a psychiatric disorder or dementia diagnosis.

## Methods

### Data

The data presented in this article are based on the databases obtained from the Federal Control and Evaluation Committee on Euthanasia that cover all officially reported cases from implementation of the law on September 23, 2002 until December 31, 2013. Euthanasia cases reported from 2014 onwards were not included in the analysis as the Committee had not yet made the data for these years available to the researchers. These databases consist of information collected from the official, standardized euthanasia registration forms sent in by the reporting physicians (see Additional file 1 for the registration form in English, authors’ translation). The data are collected by the Committee for evaluation and control purposes; the law allows that they can be made available anonymously for academic research purposes in response to a substantiated request to the Committee [3].

The databases we received provided the information on all euthanasia cases as registered by the Committee from the official registration forms. The registration form contains both open-ended and closed questions with pre-structured response categories. In the databases we received, open-ended questions such as the patient’s precise diagnosis were pre-coded into categories by the Committee. We were able to identify those cases with a psychiatric disorder or dementia because for the category ‘neuropsychiatric disorders’ the exact disorder was specified in text.

If necessary, data were recoded to obtain consistency over the years in variable coding. Cases with a combination of psychiatric and physical disorders were recoded so that they would not be included in the analysis. Inconsistencies in the data were checked and cleared with the Committee.

### Data analysis

In order to focus strictly on cases of psychiatric disorders and dementia, only those with one or more psychiatric disorders or dementia and no physical disease were included in the analysis, i.e. cases with a psychiatric disorder, such as depression, reported along with a life-threatening somatic illness such as cancer were not included. These cases were divided into four categories: 1) mood disorders, i.e. depressive disorder or bipolar disorder without somatic or other psychiatric disorders, 2) mood disorders accompanied by another psychiatric disorder, 3) other psychiatric disorders, and 4) dementia, including Alzheimer’s disease. Dementia, a progressive neurodegenerative condition, was included in the analysis because it is, according to ICD-10, classified under mental disorders (specifically in codes F00 to F03). In the summary reports issued by the Committee, dementia is included in the category of neuropsychiatric diseases along with psychiatric disorders. Issues such as the patient’s competence and the patient not being expected to die in the foreseeable future are also pertinent when evaluating these requests for euthanasia.

We used descriptive statistics to report on the annually reported number of cases with a psychiatric disorder or dementia diagnosis and the demographic, clinical and decision-making characteristics for all identified categories. Only descriptive statistics are reported, considering the low number of cases.

## Results

**Fig. 1:**
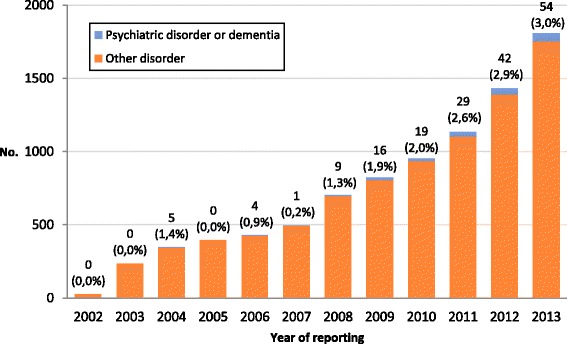
Reported cases of euthanasia in Belgium, 2002–2013*. * Numbers above the bars indicate the number of reported euthanasia cases with psychiatric disorder or dementia diagnosis and the percentage of all reported cases these numbers represent for each year

**Fig. 2:**
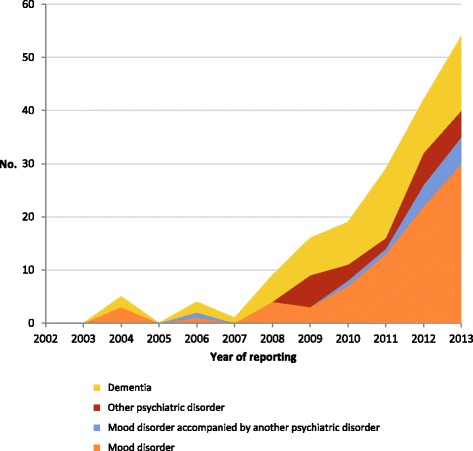
Reported euthanasia cases with a diagnosis of psychiatric disorder or dementia, 2002–2013

Between 2002 and 2013, a total of 179 cases with a psychiatric disorder or dementia diagnosis only were identified. The proportion of euthanasia cases with these disorders was 0.5% of all cases reported in the period 2002–2007, increasing from 2008 onwards to 3.0% of all cases reported in 2013 (Table 1). The increase in absolute numbers of cases with a psychiatric disorder or dementia is evident from 2008 onwards (Fig. 1), particularly in cases with a mood disorder diagnosis (Fig. 2).

**Table 1:** Reported cases of euthanasia with a diagnosis of psychiatric disorder or dementia, 2002–2013

	2002–2007	2008	2009	2010	2011	2012	2013
	No. (%)	No. (%)	No. (%)	No. (%)	No. (%)	No. (%)	No. (%)
No. (% of all reported cases)	10 (0.5)	9 (1.3)	16 (1.9)	19 (2.0)	29 (2.6)	42 (2.9)	54 (3.0)
Mood disorder	4 (40.0)	4 (44.4)	3 (18.8)	7 (36.8)	13 (44.8)	22 (52.4)	30 (55.6)
Mood disorder accompanied by another psychiatric disorder ^a^	1 (10.0)	0 (0.0)	0 (0.0)	1 (5.3)	1 (3.4)	4 (9.5)	5 (9.3)
Other psychiatric disorder ^b^	0 (0.0)	0 (0.0)	6 (37.5)	3 (15.8)	2 (6.9)	6 (14.3)	5 (9.1)
Dementia	5 (50.0)	5 (55.6)	7 (43.8)	8 (42.1)	13 (44.8)	10 (23.8)	14 (25.9)

Data presented are column percentages

^a^Mood disorder accompanied by unspecified personality disorder (5), borderline personality disorder (4), autism (1), anorexia nervosa (1), psychotic personality (1)

^b^Other psychiatric disorders were autism (6), borderline (3), posttraumatic stress disorder (2), anorexia nervosa (3), dissociative disorder (1), immature personality disorder (1), psychosis (1), anxiety disorder (1), compulsive disorder (1), paranoid schizophrenia (1), unspecified personality disorder (1), unspecified psychiatric disorder (1)

The 179 cases identified consisted mainly of mood disorders (46.4%) and dementia (34.6%), followed by other psychiatric disorders (12.3%) and mood disorders accompanied by another psychiatric disorder (6.7%) (Table 2). The majority of euthanasia cases concerned women, with percentages ranging from 58.1% in dementia to 77.1% in mood disorders. Of all the reported euthanasia cases with a mood disorder diagnosis, 38.6% concerned people aged 80 or older. The majority of reported cases concerned people less than 60 years old for mood disorders accompanied by another psychiatric disorder (83.3%) and for other psychiatric disorders (86.4%). Euthanasia most often occurred at home for those diagnosed with other psychiatric disorders (59.1%), mood disorders accompanied by another psychiatric disorder (58.3%), mood disorders (51.8%) and dementia (46.8%). Patients were expected to die in the foreseeable future in 27.4% of those with dementia, 18.2% with another psychiatric disorder, and 8.4% diagnosed with a mood disorder. Physicians most often reported unbearable psychological suffering only for euthanasia cases with mood disorders accompanied by another psychiatric disorder (83.3%), other psychiatric disorders (77.3%) and mood disorders (72.3%). The second physician consulted about the request was most often a general practitioner in cases of mood disorder (68.7%), dementia (64.5%), and other psychiatric disorder (59.1%). In cases where the patient was not expected to die in the foreseeable future, the third physician who was consulted about the request was a psychiatrist in all cases with other psychiatric disorders or mood disorders accompanied by another psychiatric disorder, in 86.8% of mood disorders and in 75.6% of dementia cases. Consultation of palliative care teams and/or additional physicians about the euthanasia request, beyond the legal requirements, ranged from 30.6% in cases with a diagnosis of dementia to 66.7% in those with a mood disorder accompanied by another psychiatric disorder.

**Table 2:** Demographic, clinical and decision-making characteristics of officially reported cases of euthanasia with a diagnosis of psychiatric disorder or dementia, 2002–2013 (*n* = 179)

	Mood disorder No. (%)	Mood disorder accompanied by another psychiatric disorder No. (%)	Other psychiatric disorder No. (%)	Dementia No. (%)
No. (row %)	83 (46.4)	12 (6.7)	22 (12.3)	62 (34.6)
Demographic characteristics				
Sex				
Male	19 (22.9)	3 (25.0)	7 (31.8)	26 (41.9)
Female	64 (77.1)	9 (75.0)	15 (68.2)	36 (58.1)
Age				
18–59	29 (34.9)	10 (83.3)	19 (86.4)	4 (6.5)
60–79	22 (26.5)	2 (16.7)	2 (9.1)	33 (53.2)
80 or older	32 (38.6)	0 (0.0)	1 (4.5)	25 (40.3)
Clinical characteristics				
Place of death				
Hospital	14 (16.9)	4 (33.3)	6 (27.3)	22 (35.5)
Home	43 (51.8)	7 (58.3)	13 (59.1)	29 (46.8)
Nursing home	23 (27.7)	0 (0.0)	1 (4.5)	10 (16.1)
Other	3 (3.6)	1 (8.3)	2 (9.1)	1 (1.6)
Patient was expected to die in the foreseeable future	7 (8.4)	0 (0.0)	4 (18.2)	17 (27.4)
Reported suffering ^a^				
Physical and psychological suffering	23 (27.7)	2 (16.7)	5 (22.7)	24 (38.7)
Only physical suffering	0 (0.0)	0 (0.0)	0 (0.0)	3 (4.8)
Only psychological suffering	60 (72.3)	10 (83.3)	17 (77.3)	35 (56.5)
Decision-making characteristics				
Type of request for euthanasia				
Current request	83 (100)	12 (100)	22 (100)	54 (87.1)
Advance euthanasia directive ^b^	0 (0.0)	0 (0.0)	0 (0.0)	8 (12.9)
Specialty of second physician ^c^				
Specialist palliative care physician	5 (6.0)	0 (0.0)	3 (13.6)	1 (1.6)
General practitioner	57 (68.7)	6 (50.0)	13 (59.1)	40 (64.5)
Specialist in the illness from which the patient suffers	21 (25.3)	6 (50.0)	6 (27.3)	21 (33.9)
Specialty of third physician if required (*N* = 151) ^d^				
Psychiatrist	66 (86.8)	12 (100)	18 (100)	34 (75.6)
Specialist in the illness from which the patient suffers	10 (13.2)	0 (0.0)	0 (0.0)	11 (24.4)
Consultations about the request beyond legal requirements				
One or more consultations	39 (47.0)	8 (66.7)	14 (63.6)	19 (30.6)
Of which with palliative care team(s)	18 (21.7)	3 (25.0)	5 (22.7)	6 (9.7)
Of which with additional physician(s)	26 (31.3)	8 (66.7)	11 (50.0)	16 (25.8)

Data presented are absolute numbers and column percentages

^a^Nature of the constant and unbearable suffering that led to euthanasia

^b^Euthanasia based on an advance euthanasia directive is only possible if the patient is in an irreversible coma

^c^The attending physician must consult a second independent physician about the serious and incurable nature of the disorder

^d^Belgian law distinguishes between those who are expected to die in the foreseeable future and those who are not expected to die in the foreseeable future. A third physician must be consulted if the patient is not expected to die in the foreseeable future. This physician should either be a psychiatrist or a specialist in the illness from which the patient suffers

All notified cases were judged to comply with the due care criteria specified in the Belgian Act on Euthanasia by the Committee.

## Discussion

Using data on all euthanasia cases officially reported in Belgium from the introduction of euthanasia legislation in 2002 until 2013, this study shows that the number and proportion of euthanasia cases with psychiatric disorders or dementia has gradually increased since 2008. Cases where any physical condition was reported by the attending physician in the euthanasia registration form were excluded from the analysis. The increase is particularly evident in cases with a diagnosis of mood disorder. However, in comparison with the total number of reported cases, euthanasia for these specific groups remains a limited practice.

Because of its controversial nature, the notable increase in euthanasia cases in people with a diagnosis of mood disorder or dementia warrants some exploration of the possible underlying reasons and significance. The trend seems to suggest that the legal possibilities of the euthanasia law are being explored more widely and have become more broadly accepted. Previous research had already shown an increase in euthanasia in groups where the practice was initially much rarer, such as those suffering from conditions other than cancer and those who are not terminally ill [17, 19]. This may reflect a typical process of change where certain groups (both patients and their physicians) slowly explore and adapt to new legal possibilities. The several years of accumulated experience with euthanasia and the transparency about each case required by the law may have caused an increased uptake of the euthanasia option in groups that were not initially considered to be the target demographic. Additionally, heightened media attention in cases that are often controversial [20] may have increased awareness among the general public of the legal possibilities in cases of psychiatric disorder or dementia. Landmark examples in Belgium, for instance, include the case of the euthanasia of Belgian writer Hugo Claus, who suffered from early Alzheimer’s disease, in 2008. That case received considerable media coverage. The acceptance by the Federal Control and Evaluation Committee for Euthanasia of certain pioneer cases as being in accordance with the law may have given patients and physicians reassurance that euthanasia in cases with a diagnosis of psychiatric disorder or dementia could be legal if all due care requirements are adhered to properly.

A large majority of Belgian physicians support the option of euthanasia for terminally ill people [21]. To our knowledge, no data are available regarding Belgian physicians’ attitudes towards euthanasia for people suffering from psychiatric disorders or dementia. A Dutch study, however, has shown that a minority of Dutch physicians find it conceivable that they would grant a euthanasia request in the case of a psychiatric disorder (34%) or early-stage dementia (40%) [22]. In the UK, the majority of physicians are opposed to changes in legislation on assisted dying, with significantly less support in the case of non-terminally ill people [23, 24].

The increase in euthanasia cases in people with a diagnosis of psychiatric disorder or dementia has given rise to some concerns, one of which relates to the specific competencies of physicians. Dealing with euthanasia requests is a challenging task for physicians, especially so when a request is based on psychological suffering [4, 9, 25]. Assessment of decision-making capacity in people with psychiatric disorders is a complex undertaking. However, studies of mental capacity in psychiatric patients show that mental capacity can be reliably assessed [26, 27]. It is not possible, however, to determine whether these assessments were used as this information was not available in the Committee’s databases. Further, consensus about the meaning of medical futility in the context of psychiatry is lacking [28] and long-term outcomes of psychiatric illness are complicated to determine [29, 30]. Despite all existing and novel treatments for mood disorders [31–35], euthanasia may still be the only option available for certain people suffering from severe treatment-resistant depression. Given the complex nature of euthanasia requests expressed by people with mental illness, it is essential to develop practice guidelines for evaluating and responding to these requests. In 2004 the Dutch Psychiatric Association issued a guideline for application of the euthanasia law in psychiatric practice [36], but no official guideline is available in Belgium.

A second concern relates to the vulnerability of this patient population. People with chronic mental conditions are considered to be a vulnerable population, particularly in the context of assisted dying. As a wish to die can be a symptom of mood disorder, an area of tension arises between respecting the patient’s autonomy on the one hand and suicide prevention and harm reduction on the other [37]. Further, a rather large proportion (38.8%) of euthanasia cases in mood disorders in our study were in people aged 80 or older. This finding differs from a recent study of 100 people suffering from a psychiatric disorder who requested euthanasia, which found that most cases involved younger people [38]. However, our finding is consistent with a study examining psychiatric euthanasia and assisted suicide cases in the Netherlands [39]. Older people have an increased risk of having lost a partner, of experiencing social isolation or of the accumulation of chronic physical conditions associated with old age, which are in turn risk factors for depression and are associated with developing a wish to die [40–42]. However, research also showed that a majority of older respondents with a wish to die suffered from depressed mood without meeting the diagnostic criteria to qualify for a depressive disorder [40, 41]. This emphasizes the importance of careful assessment of euthanasia requests expressed by this population.

A third concern relates to the procedures used to evaluate euthanasia requests in persons with psychiatric disorders or dementia. Considering the potential effect of mental illness on decision-making capacity, the possibility exists that the desire to die is a symptom of the disorder, and, since the prognosis is difficult to make, questions can be raised regarding the need for supplementary monitoring of cases involving people with psychiatric disorders or dementia. For instance, some have suggested additional monitoring through the appointment of a separate subcommittee to review and control these specific cases or through a priori control of euthanasia requests based on unbearable suffering resulting from a psychiatric disorder. Although this procedure may not be desirable for terminally ill people as it can create unnecessary delay, it may be relevant to consider it for requests expressed by people diagnosed with a psychiatric disorder or dementia.

Although it is a legal requirement to do so, a psychiatrist was not consulted in all cases with a diagnosis of psychiatric disorder. A possible explanation for this is that physicians may have only mentioned the diagnosis that was the main cause of the unbearable suffering; it may be that in these cases the person suffered from multiple pathologies, in which cases the Committee agreed that the legally required third physician could be a general practitioner.

Surprisingly, the reporting physician indicated in a number of cases with a psychiatric disorder diagnosis that the patient was expected to die in the foreseeable future. The Committee defines this as when death can be expected within the next few days, weeks or months, which implies that additional procedural requirements have to be followed in cases of non-progressive or slowly evolving disorders [18], which includes psychiatric disorders. However, if the patient was expected to die in the foreseeable future, but two physicians were consulted about the euthanasia request and the one-month waiting time was respected, the Committee deems these cases in accordance with the law. A possible explanation for our finding is that these people were severely weakened as a consequence of the psychiatric disorder or dementia they were suffering from, leading to death being expected in the near future. An alternative explanation is that the person also suffered from a terminal condition not registered in the database or in the registration form; the reporting physician may have only mentioned the condition that led to the euthanasia request and not the presence of another advanced chronic illness that was not itself the reason for the request. Another possibility is that ‘in the foreseeable future’ is interpreted broadly by the reporting physician; it is also possible that the physician may have expected the patient to commit suicide in the near future. These cases, although there are only a few of them, illustrate that the evaluation of euthanasia requests from people with serious mental illness may require a different or more complex procedure.

One strength of this study is the use of data based on routinely collected information from the official, standardized euthanasia registration forms; the Committee contacted the reporting physicians when important information was missing from the registration form. Another is that we studied all reported cases of euthanasia with a psychiatric disorder diagnosis in an entire jurisdiction since the implementation of the Belgian Act on Euthanasia in 2002, making it possible to study year-by-year trends.

The study also has limitations. The data were gathered for review and control purposes and coded by the Committee. Certain information from the registration form that could provide more detailed insights into the characteristics and decision making of the selected euthanasia cases was not recorded in the databases, e.g. the reasons why the patient’s suffering could not be alleviated or the patient’s treatment history. Furthermore, only cases reported to the Committee could be analysed and not those which were unreported. Due to the complex and controversial nature of euthanasia in cases involving a psychiatric disorder or dementia, it is possible that not all were reported, especially in the earlier years after legalization [43]. Furthermore, as there is a requirement to report a euthanasia request which is carried out but not one which is not, we had no information on the number of actual requests for euthanasia coming from those who suffer from psychiatric disorders or dementia. Therefore, it is not possible to report on the number of requests granted, refused or withdrawn.

## Conclusions

While euthanasia on the grounds of unbearable suffering caused by a psychiatric disorder or dementia remains a relatively limited practice in Belgium, its prevalence has risen since 2008. If, as this study suggests, people with psychiatric conditions or dementia are increasingly seeking access to euthanasia, the development of practice guidelines is all the more desirable if physicians are to respond adequately to these highly delicate requests.

## Additional file

Additional file 1: Euthanasia registration form. (PDF 115 kb)
