# Supplementary material for: Euthanasia for people with psychiatric disorders or dementia in Belgium: analysis of officially reported cases
Source: BMC Psychiatry. 2017 Jun 23;17:203. doi: 10.1186/s12888-017-1369-0 (PMC5481967; doi:10.1186/s12888-017-1369-0)
Supplement: Additional file 1: — Euthanasia registration form. (PDF 115 kb) [file 12888_2017_1369_MOESM1_ESM.pdf]

Appendix

Euthanasia registration form

REGISTRATION FORM EUTHANASIA

Form to be sent by registered mail with a proof of receipt to the Federal Control and Evaluation Committee for the application of the Act on Euthanasia, within four working days, to the following address:

Federale Controle- en Evaluatiecommissie Euthanasie
Victor Hortaplein 40 bus 10 (verdieping 7C)
1060 Brussel

References to articles of law in this document relate to the Act on Euthanasia of 28 May 2002 (Belgian Official Gazette of 22 June 2002).

TO AVOID CONFUSION

IN ACCORDANCE WITH THE LAW ON EUTHANASIA THE REGISTRATION FORM MAKES A DISTINCTION BETWEEN EUTHANASIA BASED ON A ‘REQUEST FOR EUTHANASIA’ AND EUTHANASIA BASED ON AN ADVANCE ‘DIRECTIVE’.

THE REQUEST FOR EUTHANASIA CONCERNS THE REQUEST OF AN ILL PATIENT, WHO IS IN A MEDICALLY FUTILE CONDITION OF CONSTANT AND UNBEARABLE PHYSICAL OR MENTAL SUFFERING THAT CANNOT BE ALLEVIATED, RESULTING FORM A SERIOUS AND INCURABLE DISORDER CAUSED BY ILLNESS OR ACCIDENT (ART 3).

ON THE OTHER HAND, AN ADVANCE DIRECTIVE IS USED TO REQUEST EUTHANASIA BEFOREHAND, IN CASE ONE, AT A LATER TIME IN LIFE, WOULD END UP IN A STATE OF UNCONCIOUSNESS AND THIS CONDITION WOULD BE IRREVERSIBLE AND ONE WOULD SUFFER FROM A SERIOUS DISORDER CAUSED BY ILLNESS OR ACCIDENT (ART 4).

PART 1

Personal information relating to the patient, the physician, the consulted physicians and others.

This part is strictly confidential. <u>It must be sealed by the physician and can only be opened by decision of the committee.</u> Under no circumstance can it be used for the assessment task of the committee on behalf of the legislative Chambers.

1. the patient
1.1. surname:
1.2. first name:
1.3. place of residence:

2. the physician
2.1. surname:
2.2. first name:
2.3. registration number RIZIV[1]:
2.4. place of residence:
2.5. e-mail

3. physician(s) whose consultation is required by law
3.1. second physician (in each case, art. 3§2,3° and art. 4§2,1°)
surname:
first name:
place of residence:
registration number RIZIV:
date of consultation:
3.2 in case the physician is of the opinion that the patient is not expected to die in the foreseeable future, a third consulted physician (art. 3§3,1°)
surname:
first name:
place of residence:
registration number RIZIV:
date of consultation:

[1] RIZIV: Rijksinstituut voor ziekte- en invaliditeitsverzekering, National Institute for Health and Disability Insurance

4. any others who were consulted (as stipulated in art. 3§2, 4°-6° and art. 4§2, 2°-4°)

4.1. surname:

first name:

capacity:

place of residence:

date of consultation:

4.2. surname:

first name:

capacity:

place of residence:

date of consultation:

4.3. surname:

first name:

capacity:

place of residence:

date of consultation:

4.4. surname:

first name:

capacity:

place of residence:

date of consultation:

4.5. surname:

first name:

capacity:

place of residence:

date of consultation:

4.6. In case of euthanasia based on an advance euthanasia directive

surname of the designated first proxy:

first name:

date of consultation:

surname of the designated second proxy:

first name:

date of consultation:

DATE, PHYSICIAN'S SIGNATURE AND STAMP

PART 2

Conditions and procedure under which euthanasia was performed.
This part of the document is strictly confidential; it will serve to allow the committee to verify whether the euthanasia was performed according to the conditions and procedures stipulated in the law.
It cannot contain any names (such as the name of the patient, the physician, institution, etc.).

1. the patient (do not mention name)
   1.1. place and date of birth: . . / . . / . .
   1.2. sex:

2. date of death: (d/m/y) . . / . . / . .
   hour of death:
   place of death (tick)
   - ☐ home
   - ☐ hospital
   - ☐ nursing home
   - ☐ other

3. **precise diagnosis**:

**In case euthanasia was performed on a patient who was not conscious, based on an advance euthanasia directive, skip items 4 up until 12 and proceed to item 13.**
4. nature and description of the constant and unbearable suffering:
5. reasons why this suffering could not be alleviated:
6. elements proving that the request was voluntary, well-considered and repeated, and did not originate from external pressure:
7. can it be assumed that the patient would have died in the foreseeable future?<br>yes ☐ no ☐
8. procedure followed by the physician (art. 3) (to be ticked and completed if followed)<br>☐ existence of a euthanasia request in writing (art. 3§4)<br>date of the request: . . / . . / . .<br>☐compiled, dated and signed by the patient<br>or<br>☐compiled, dated and signed, in the presence of a physician, by an adult third party chosen by the patient without material interest in the death of the person concerned<br>☐mentioned the reasons why the patient was unable to put the request in writing and sign<br>☐ informed the patient of his/her health status and life expectancy (art. 3§2, 1°)<br>☐ discussed the request for euthanasia with the patient (art. 3§2, 1°)<br>☐ informed the patient about remaining therapeutic options and consequences (art. 3§2, 1°)<br>☐ informed the patient about palliative care and consequences (art. 3§2, 1°)<br>☐ established persistent physical or psychological suffering of the patient (art. 3§2, 2°)<br>☐ established that the request for euthanasia was repeated (art. 3§2, 2°)<br>☐ discussed the request for euthanasia with members of the nursing team (art. 3§2, 4°)<br>☐ discussed the request for euthanasia with relatives designated by the patient (art. 3§2, 5°)<br>☐ ensured that the patient discussed the request for euthanasia with the desired people (art. 3§2, 6°)<br>☐ recorded the course of the followed procedure and the written documents in the medical file (art. 3§5)

9. Independent physicians who were consulted as legally required (do not mention identity)

9.1 the second physician (art. 3§2, 3°)

physician's specialisation:

date of consultation: . . / . . / . .

recommendation of the consulted physician (according to his written report) regarding **the serious and incurable character of the disorder and the constant and unbearable suffering that cannot be alleviated**:

9.2 if necessary, the third physician in case **the patient's death is not expected in the foreseeable future** (art. 3§3, 1°)

physician's specialisation:

date of consultation: . . / . . / . .

recommendation of the consulted physician (according to his written report) regarding **the constant and unbearable suffering that cannot be alleviated** and the **voluntary, well-considered and repeated character of the request**

10. other persons or authorities consulted (do not mention identity):

10.1 capacity:
date of consultation:

10.2 capacity:
date of consultation:

10.3 capacity:
date of consultation:

10.4 capacity:
date of consultation:

10.5 capacity:
date of consultation:

11. the manner and drugs used in performing euthanasia:

12. additional information the physician wishes to impart:

The following items 13 up until 19 concern euthanasia cases involving a patient who WAS NOT CONSCIOUS, based on an advance euthanasia directive.

13. ☐ existence of a properly formatted advance directive according to the model determined by Royal Decree of April, 2 2003
date of the document: . . / . . / . .
☐ compiled, dated and signed by the patient
☐ compiled, dated and signed, in case the patient was physically unable, by an adult third party chosen by the patient without material interest in the death of the person concerned
☐ the reasons why the patient was unable to put the request in writing and sign are mentioned
☐ a medical certificate is enclosed vouching for the impossibility (of the patient to sign)
☐ one or more proxies were designated
☐ the course of the followed procedure and the written documents are recorded in the medical file (art. 4§2, 4°)

14. ☐ the unconscious state of the patient was irreversible

15. independent physician consulted (art. 4§2, 1°):
physician's specialisation:
date of consultation: . . / . . / . .
physician's recommendation concerning the patient's irreversible medical condition:

16. ☐ discussion with the proxies designated in the advance directive (art. 4§2, 3°)
☐ discussion with the nursing team (art. 4§2, 2°)
☐ discussion with the patient's relatives, designated by the proxies (art. 4§2, 4°)

17. any other persons or instances consulted **(do not mention name)**

17.1 capacity:
date of consultation

17.2 capacity:
date of consultation

17.3 capacity:
date of consultation

17.4 capacity:
date of consultation

18. the manner and drugs used in performing euthanasia:

19. additional information the physician wishes to impart:
